# Useful design of custom-made mouthguard for athletes undergoing orthodontic treatment with brackets and wires

**DOI:** 10.1016/j.jds.2021.03.010

**Published:** 2021-04-09

**Authors:** Ruman Uddin Chowdhury, Hiroshi Churei, Gen Tanabe, Yuriko Yoshida, Kairi Hayashi, Hidekazu Takahashi, Takahiro Wada, Motohiro Uo, Takahiro Mizobuchi, Nafees Uddin Chowdhury, Toshiaki Ueno

**Affiliations:** a Department of Orthodontics, Dental Unit, Kumudini Women's Medical College, Dhaka University, Dhaka, Bangladesh; b Department of Sports Medicine/Dentistry, Graduate School of Medical and Dental Science, Tokyo Medical and Dental University, Tokyo, Japan; c Department of Special Care Dentistry, Hiroshima University, Hiroshima, Japan; d Department of Oral Biomaterials Development Engineering, Graduate School of Medical and Dental, Tokyo Medical and Dental University, Tokyo, Japan; e Department of Advance Biomaterial, Graduate School of Medical and Dental, Tokyo Medical and Dental University, Tokyo, Japan; f Harimayabashi Mizobuchi Dental Clinic, Kochi, Japan

**Keywords:** Mouthguard, Orthodontics, Shock absorption

## Abstract

**Background/purpose:**

Custom-made mouthguards (MGs) are strongly recommended for athletes during sports activities to prevent dental injuries. Athletes undergoing orthodontic treatment and wearing brackets require specially designed MGs for better protection and to create more space that will not hinder the planned orthodontic tooth movement. The purpose of this study was to fabricate effective, specially designed, custom-made MGs for patients or athletes with ongoing orthodontic treatment and to evaluate the shock absorption abilities of these MGs by an *in vitro* comparison of three different designs.

**Materials and methods:**

Three different types of specially designed, double-layered MGs, (i) creating inter bracket space inside the MG, (ii) embedding silicon wax inside the MG, and (iii) creating a buffer space with additional hard insertion, were fabricated from a simulated bracket attached model. Impact test was performed using a free-falling object on a vertical rod, and the strain-gauge system was used to assess the strain on the dentition with the MGs for the comparison of the shock absorption abilities of the three types. Analysis of variance at a significance level of 5% and multiple comparisons were performed for statistical analysis.

**Results:**

The strains on the dentition with the MG creating buffer space with hard insertion were significantly lower than those with the other two types of MG (P < 0.001).

**Conclusion:**

Insertion of a hard material and ensuring buffer space between the MG and the teeth and/or appliance was more effective than other methods of fabricating custom-made MGs to prevent sports-related traumatic dental injuries in athletes undergoing orthodontic treatment.

## Introduction

Mouthguards (MG) play an important role in preventing oral injuries and preserving the oral structures of athletes during contact and non-contact sports.^1^, ^2^, ^3^ MGs can considerably reduce the risk of displacement, fracture, and/or avulsion of teeth, and fracture and/or concussion of the alveolar processes, condyles, and/or body of the mandible.^4^^,^^5^ The Oral Health Foundation has advised that an MG should be worn at all times while participating in any contact sport,^6^ and the American Dental Association has listed specific sports that require the use of an MG (Table 1).^1^ The American Society for Testing and Materials has classified MGs into three basic types: stock, mouth-formed or boil-and-bite, and custom-made. Custom-made MGs are the preferred choice for players and are recommended by the Fédération Dentaire International-World Dental Federation.^7^

**Table 1:** Sports for which ADA advised use of mouthguard.

Acrobatics	Equestrian events	Ice hockey	Shot put	Squash
American football	Extreme sports	Inline skating	Skateboard	Surfing
Baseball	Field events	Lacrosse	Skiing	Volleyball
Basketball	Field hockey	Martial art	Sky diving	Water polo
Bicycling	Gymnastics	Racquetball	Soccer	Weightlifting
Boxing	Handball	Rugby	Softball	Wrestling

Though previous research on MGs and oral trauma were not specifically conducted among participants exhibiting a certain type of occlusion (normo-occlusion or malocclusion), patients or athletes with malocclusion are known to be more susceptible to dental injuries, specifically in the maxillary anterior region because of large overjet and/or short upper lip, depending on the type of malocclusion present.^8^^,^^9^ Ironically, patients or athletes with malocclusion undergoing fixed orthodontic treatment exhibit more than normal overjet because of the presence of brackets. Thus, athletes undergoing fixed orthodontic treatment may show even higher risks of sustaining dental traumatic injuries,^10^ loosening of brackets, and/or deformation of orthodontic wires caused by blows to the appliances.^11^ Several researchers have suggested the technique of fabricating specially designed MGs for athletes with malocclusion undergoing fixed orthodontic treatment.^12^, ^13^, ^14^, ^15^, ^16^, ^17^ Bonded orthodontic brackets pose problems in the use of vacuum-formed MGs because of the movement of the teeth during the course of orthodontic therapy. Previous studies have suggested blocking the labial/buccal surfaces of the teeth on working models during the fabrication of MGs to create space for planned tooth movements.^13^^,^^15^^,^^17^, ^18^, ^19^

In 1997, Yamada et al. first described the process of fabricating a custom-made MG that addressed this issue by creating space along the inner layer of the MG.^15^ They suggested that the impression should be made after the brackets and the arch wires are covered with silicone rubber, followed by the fabrication of a custom-made MG from the obtained model. In 2004, Croll et al. outlined a similar method that used a thin layer of utility wax to cover the brackets of the anterior teeth before making the impression. In the obtained dental stone model, the brackets and wire areas were covered using the window sealing material, mortite, in the regions of anticipated tooth movement to avail space inside the MG.^17^ Mortite or putty-like silicone material is recommended for blocking out space, since the materials used should be heat-resistant as considerable heat is generated during the vacuum-forming process.^19^ Pacheco et al., in 2010 described that polyvinylsiloxane impression material was adapted around the brackets and arch wires during impression-making, and a 1.5-mm layer of light-polymerized acrylic resin was adapted on the plaster model over the brackets and areas where individual teeth were expected to move.^18^ Additionally, they suggested the removal of the inner layer of the fabricated MG to create more space that will not hinder the planned orthodontic tooth movement. Such specially designed MGs should not interfere with the movements of the teeth, not cause undue friction, and should allow the sliding mechanics of the brackets along the arch wires. However, the ideal process of fabricating specially designed custom-made MGs that would enhance the shock absorption ability is yet to be determined.

The purpose of this study was to fabricate effective, specially designed, custom-made MGs for patients or athletes with ongoing orthodontic treatment and to evaluate the shock absorption abilities of these MGs by an *in vitro* comparison of three different designs.

## Materials and methods

### Fabrication of the maxillary model with simulated brackets

**Figure 1:**
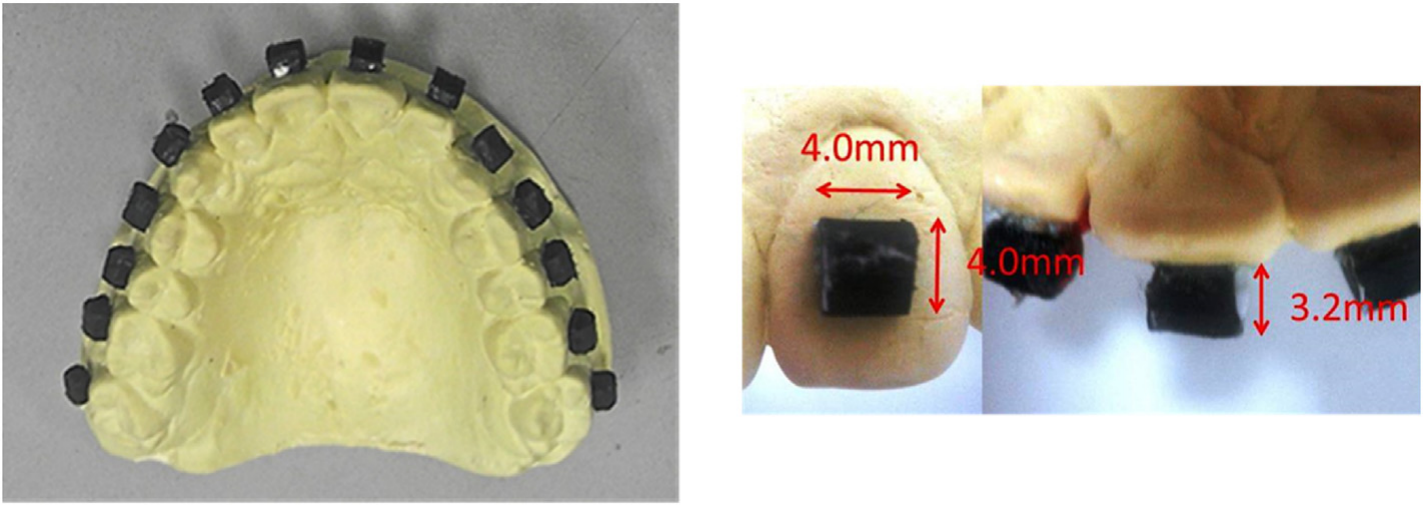
Plaster model with Aquaplast Watercolor blocks (simulated bracket).

An alginate impression of the maxillary arch of a patient with normal occlusion was recorded to fabricate a maxillary model. The primary model was fabricated from the impression using anhydrite plaster (New Plastone; GC Corp., Tokyo, Japan). A 3.2-mm thick sheet of a commercial thermoplastic splinting material, Aquaplast Watercolors (AP; Polycaprolactone material; Homecraft Rolyan, Huthwaite, North Nottingham, UK), was cut into a rectangular block of dimensions 4 mm × 4 mm × 3.2 mm (vertical × horizontal × anteroposterior length) using an ultrasonic cutter (Labo Sonic Cutter model NE87; Nakanishi Inc., Tochigi, Japan). Fourteen AP blocks, 1 mm larger than the actual bracket size, were prepared to simulate orthodontic brackets and were attached to the labial/buccal surfaces from the maxillary left second molar to right second molar of the primary model by a commercial adhesive (Aron alpha Pro No.5, Toagosei, Tokyo, Japan) (Fig. 1). The correct positions of the simulated brackets along the long axes of the teeth were ensured considering the distance from the incisal/occlusal edges of the teeth to the center of the AP block according to the planned bracket-fixing point (Table 2). The simulated brackets were placed on the primary model according to Dr. Boone's bracket placement method, a commonly used bracket positioning system with mesiodistal and angular accuracy.^20^ An impression of the primary model was recorded using silicone impression material (Duplicon; Shofu Inc, Kyoto, Japan), and a secondary model was fabricated using synthetic denture base resin (Palapress vario; Heraeus Kulzer GmbH, Hanau, Germany). The primary model was used for the fabrication of the MGs, and the secondary resin model was used for conducting the impact test, as resin can withstand heavy forces from a free-falling object. All sides of the brackets were trimmed in the secondary resin model by approximately 1 mm so that the actual dimensions of the brackets could be simulated. This also provided space inside the MG after its fabrication at a later stage.

**Table 2:** The distance from the incisal/occlusal edges of the teeth to the center of the AP block according to the planned bracket-fixing point (AP: Aquaplast Watercolors).

Tooth	1	2	3	4	5	6	7
Distance (mm) (from incisal/occlusal edge)	4.5	4	5	4.5	4.5	4.5	4.5

### Fabrication of three different types of specially designed double-layered mouthguards

**Figure 2:**
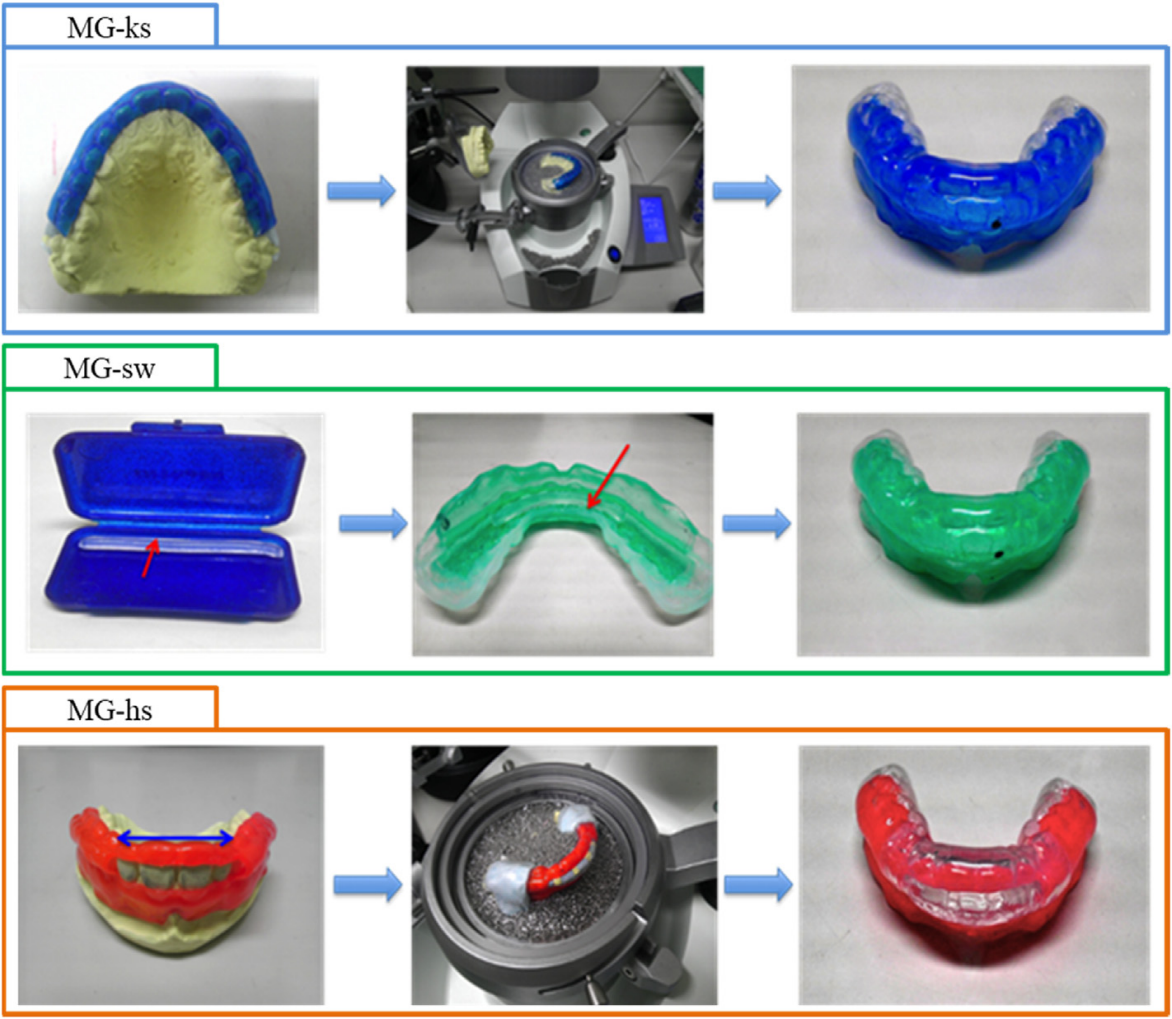
Three different types of specially designed, double-layered mouthguards (MGs) (MG-ks: Blue): MG-keeping space; First layer from the maxillary right first molar to left first molar; second layer the maxillary left second molar to right second molar. (MG-sw: Green): MG with silicon wax; Silicon wax embedded inside the MG covering from the maxillary right canine to left canine. (MG-hs: Orange): Hard-sheet MG; polyethyleneterephthalate-glycol was added on the labial surface of the first layer (left), and the second layer was fabricated over the first layer (right).

Polyolefin sheets (MG21™, CGK Corp., Hiroshima, Japan) were chosen for the fabrication of the MGs. The first layer was fabricated using a 2-mm thick round sheet and the second layer using a 3-mm sheet to fabricate a double-layered MG. The following three types of special MGs were fabricated (Fig. 2).

(i) An MG-keeping space (MG-ks; Blue) was fabricated from the primary plaster model after the AP blocks were attached to the teeth on the casts. The inter bracket spaces were sealed using laboratory silicon impression material (Lab Silicon; Shofu, Kyoto, Japan) from the maxillary left second molar to right second molar. The first layer (inner layer) was fabricated using the 2-mm thick, blue-colored MG sheet by placing the primary plaster model on the stage of the vacuum-forming machine (Erko-form-3D; Erkodent Erich Kopp GmbH, Pfalzgrafenweiler, Germany). After cooling, the inner layer was trimmed to retain the parts corresponding to the labial and buccal areas from the maxillary left first molar to right first molar (Fig. 2, left). Similarly, the second layer was fabricated by thermoforming the 3-mm thick clear sheet over the first layer extending from the maxillary left second molar to right second molar (Fig. 2, right).
(ii) The first layer (inner layer) of an MG with silicon wax (MG-sw; Green) was fabricated following the same procedure using a 2-mm thick, green-colored sheet covering the labial and buccal surfaces from the maxillary left first molar to right first molar. The second layer was fabricated by thermoforming a 3-mm thick clear sheet over the first layer, extending from the maxillary left second molar to right second molar. Silicone wax (ORTHO-SIL™, TOMY International, Tokyo, Japan) was then carefully embedded inside the first layer from the labial surface of the maxillary left canine to right canine (Fig. 2).
(iii) A hard-sheet MG (MG-hs; Orange) was fabricated by the same procedure using an orange-colored sheet for the fabrication of the first layer. The anterolabial part of the MG from the maxillary left lateral incisor to right lateral incisor was excised using an ultrasonic cutter. A 1-mm thick layer of clear polyethyleneterephthalate-glycol (PET-G)-based hard, commercial, medical, orthodontic splint material (Erkodur, Erkodent Erich Kopp GmbH) was attached over the first layer extending from the maxillary left lateral incisor to right lateral incisor. The PET-G layer was softened using a heating gun (Hakko Heating Gun 880B, Hakko Corporation, Osaka, Japan), the air flow of which is controllable, with a velocity of 330 m/min, air flow of 0.18 m^3^/min, and maximum temperature of 400 °C,^21^ and was attached to the first layer of the MG on the labial surfaces from the maxillary left canine to right canine. The PET-G material adheres to the polyolefin sheet as both are polyethylene-polypropylene co-polymer thermoplastic materials. The second layer of 3-mm thick clear sheet was thermoformed over the first layer, extending from the maxillary left second molar to right second molar (Fig. 2).

**Figure 3:**
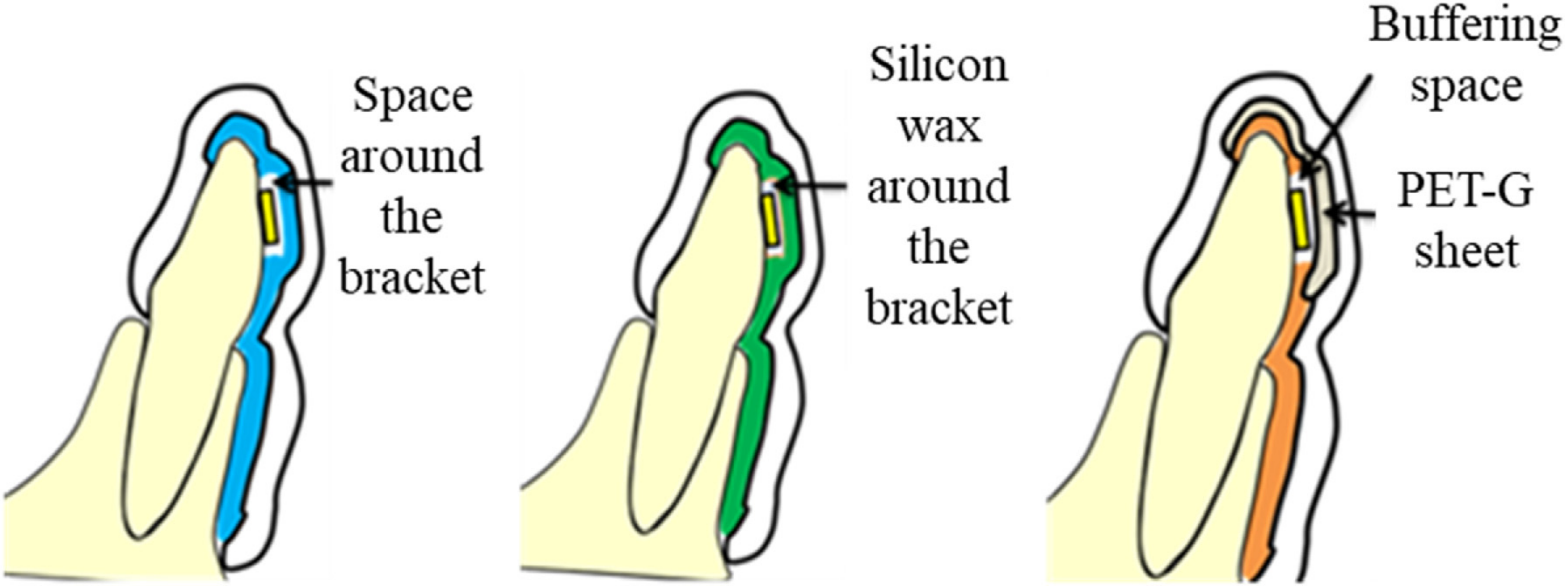
Schematic drawing of the three different types of mouthguards (MGs). MG-ks (Blue): MG-keeping space; MG-sw (Green): MG with silicon wax; and MG-hs (Orange): Hard-sheet MG.

All MGs were cut, trimmed, and polished such that the labial and occlusal surfaces attained the thicknesses of approximately 3 mm for the drop impact test, which was conducted with strain gauges, to compare the shock absorption abilities of the three types of MGs. Fig. 3 shows a schematic representation of the three different moldings of the MGs on the resin model.

### The drop impact test

**Figure 4:**
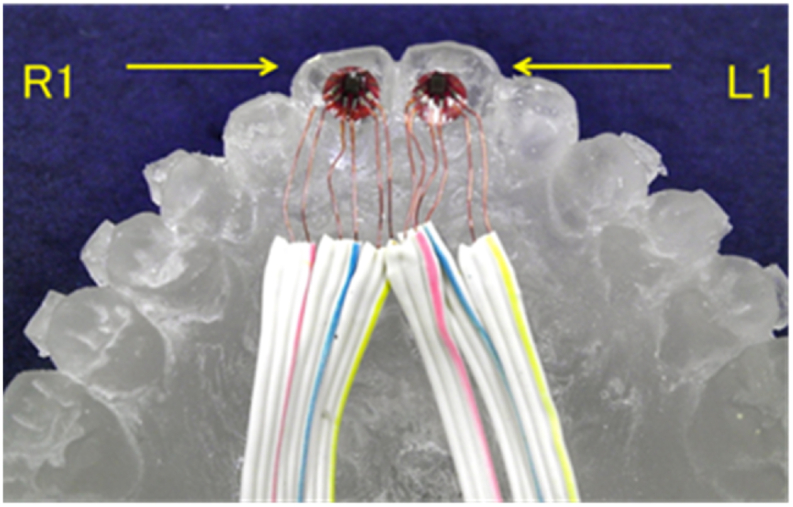
Strain gauge on the palatal surface of the maxillary central incisors.

The drop impact test was performed on the resin model with the MGs. Strain gauges (KFG-1-120-D17-11L5M3S (φ 5 mm), Kyowa Electronic Instruments Co., Tokyo, Japan) were attached to the palatal surfaces of the left and right central incisors on the resin model by adhesive cement (CC-35, Kyowa Electronic Instruments Co.) (Fig. 4). Each MG was just wearing on the resin model after attaching strain gauges. The other ends of the strain-gauge wires were fixed to channels (Ch-r: right side R1, Ch-l: left side L1) of the sensor interface PCD-300A (Kyowa Electronic Instruments Co.).

**Figure 5:**
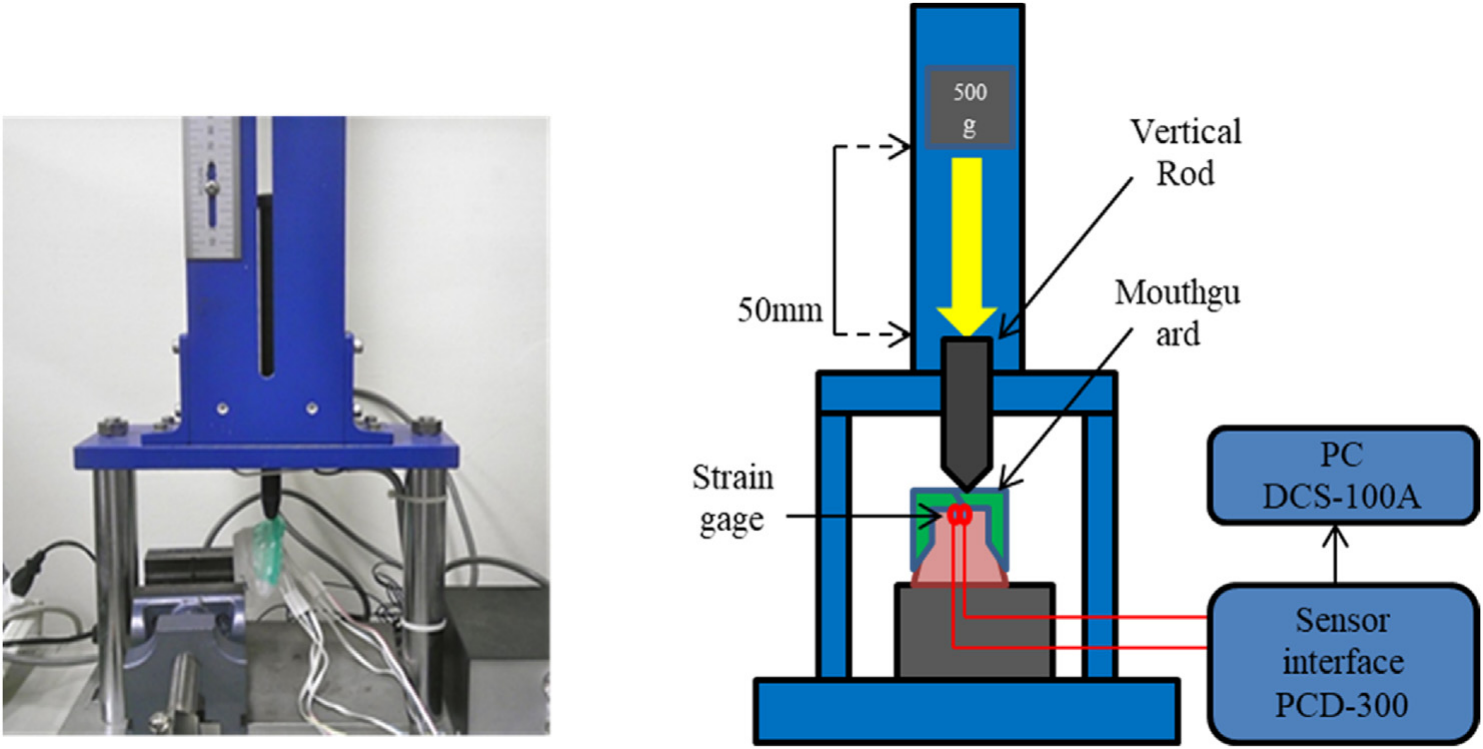
Impact test on the mouthguard (left) and schematic drawing of the impact test (right).

After preparing the strain gauges, the resin model with the MG was placed on the stainless-steel platform of the impact-testing machine (modified IM-201, Tester Sangyo Co. Ltd, Saitama, Japan), and a vertical rod with a blunt edge (a 3/16-inch-diameter rounded end) was set on the central incisors one at a time. The impact load of a free-falling 500-g object from a height of 5 cm was applied separately to the labial surfaces of the right and left central incisors on the model with the MG via the rod (Fig. 5) (R, L).^22^ Changes in strain data (R1 and L1), during and after the application of the impact load on MG-ks, MG-sw, and MG-hs were recorded using the sensor interface and were transferred to a personal computer at a sampling rate of 2000 Hz. Data obtained from the two strain gauges (right and left central incisors) were controlled by the data acquisition software DCS-100A (Kyowa Electronic Instruments Co.), and each MG was analyzed five times. The maximum principal stress and strain obtained from the MGs were calculated from both R1 and L1 channels.

The maximum principal strain (ε_max_), while using such strain gauges, is defined as follows:

$$\varepsilon_{\max}=\frac{1}{2}\left[\varepsilon \text{a}+\varepsilon \text{c}+\sqrt{2\left\{{\left(\varepsilon a-\varepsilon b\right)}^{2}+{\left(\varepsilon b-\varepsilon c\right)}^{2}\right\}}\right]$$

The strain gauge used in this study has three-axis measurements at 0°, 45°, and 90°.

εa: strain measured at the 0° axis, εb: strain measured at the 45° axis, εc: strain measured at the 90° axis.

To confirm the impact load, the same drop impact was applied without model and MG on a stainless-steel platform placed on three dynamic LMB-A-2KN compression load cells with a rated capacity of 2 kN (Kyowa Electronic Instruments Co.), which were located 120° apart on the platform of the impact tester. Changes in force during the impact test were recorded on a personal computer through an EDX-100A amplifier (Kyowa Electronic Instruments Co.).^22^ The intensity of the first peak of the sum of the measured forces was calculated as the impact load.

### Statistical analysis

The obtained data were analyzed using statistical software JMP14 (SAS Institute Inc., Cary, NC, USA). The stresses on the maxillary incisors of the dental model with the MGs were statistically compared among the three types of MGs using analysis of variance at a significance level of 5% and Tukey's honest significant difference, because the data were normal and homoscedastic (Shapiro–Wilk normal test; P = 0.25, and Levene test; P = 0.28). The strains were statistically compared among the three types of MGs using analysis of variance at a significance level of 5% and Steel–Dwass test, because the data were not normal or homoscedastic (Shapiro–Wilk normal test; P < 0.05, and Levene test; P < 0.05).

## Results

**Figure 6:**
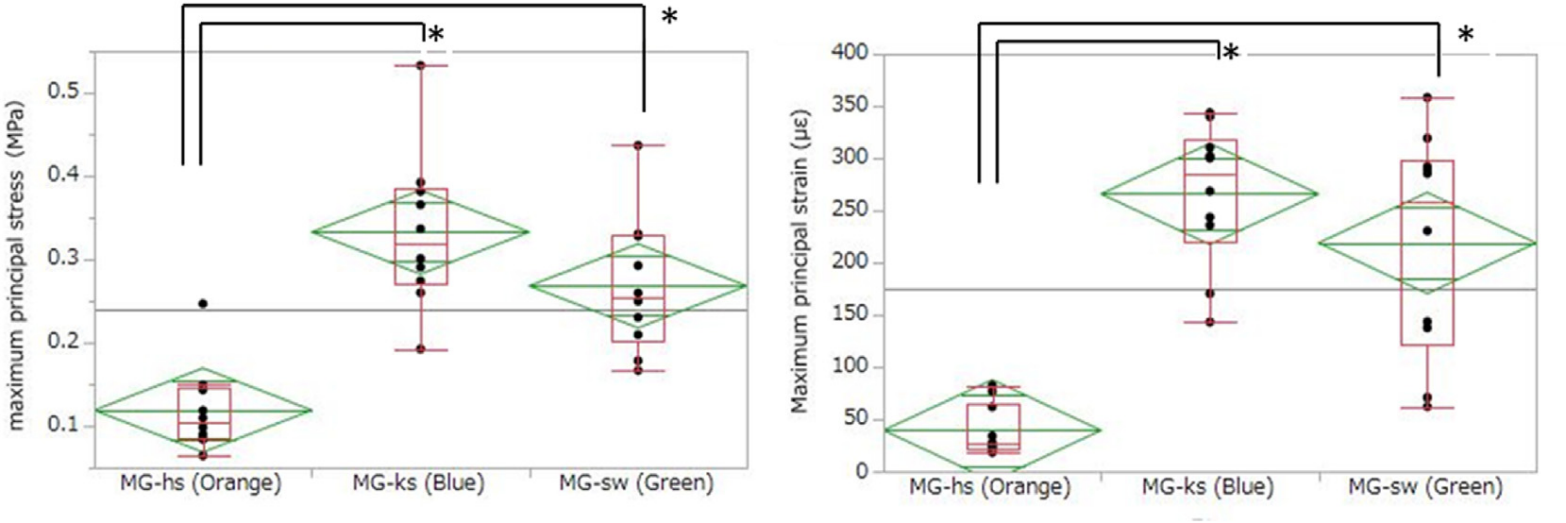
The results of the stresses test (Left) and strains test (Right) (∗P < 0.001).

The stresses and strains on all MGs are shown in Table 3 and Fig. 6. The object falling from a height of 5 cm on the rod generated a force of 1664 N ± 49 N on the metal platform of the impact-testing machine without model and MG as the impact load. The maximum principal stress on MG-hs was significantly lower than that on MG-ks and MG-sw (P < 0.001 and P < 0.001, respectively), and no significant difference was observed between MG-ks and MG-sw (P = 0.17).

**Table 3:** The results of the stresses and strains test.

	Maximum principal strain (με ± SD)	Maximum principal stress (MPa ± SD)
	R1 + L1	R1 + L1
MG-ks (Blue)	266.0 ± 68.0	0.333 ± 0.093
MG-sw (Green)	219.0 ± 107.0	0.268 ± 0.082
MG-hs (Orange)	39.5 ± 24.5	0.119 ± 0.052

MG: Mouthguard

MG-ks: MG-keeping space, MG-sw: MG with silicon wax, MG-hs: hard-sheet MG.

Comparisons of maximum principal strains showed that MG-hs exhibited significantly lower strain than MG-ks and MG-sw (P < 0.001 and P < 0.001, respectively), and no significant difference was observed between MG-ks and MG-sw (P = 0.56).

## Discussion

Specially designed MGs for athletes with malocclusion while wearing orthodontic appliances are indispensable. With regard to orthodontic biomechanics, previous studies have proven that the use of protectors (plastic or resin-based) can increase the friction between the wire, ligature, and brackets.^23^, ^24^, ^25^, ^26^ Ideally, materials used over brackets should not interfere or even contact the wire and bracket slot during orthodontic tooth movement. However, another study showed that customized thermoplastic protectors that cover the brackets do not differ significantly in friction, as compared to controls without protectors.^27^ Considering these key points, we fabricated thermoplastic custom-made MGs allowing adequate space for the wires and brackets.

All MGs comprised two layers of block-out space. In case of MG-sw, a layer of silicone wax was added on the inner surface of the MG for experimental purposes. In case of MG-hs, additional space was created by inserting hard PET-G. All MGs showed consistent final thicknesses of 3 mm on the labial side for accurate comparison. Most *in vivo* studies or case reports have mentioned the use of heat-resistant mortite or laboratory silicone impression material for blocking out the space and achieving adequate thickness.^15^^,^^17^^,^^18^ However, Pacheco et al. suggested the removal of the first inner layer to create additional space for orthodontic tooth movement.^18^ If this protocol is followed, the final thickness of the MG would not be consistent, according to the ideal requirements.

To replicate the clinical situation, all MGs in this study were fabricated such that they did not hinder tooth movement or require adjustment for weeks. MG-hs consisted of additional space and hard sheets were inserted. Such MGs allow tooth movement along the arch wire in the mesiodistal direction and anterior tipping movements and torque in the labial direction, as they exhibit additional anterior buffer space. However, readjustments might be required to facilitate extrusion or palatal movement of the teeth.

The results of this study show that the newly designed MG with additional space and hard-insert (MG-hs) showed better shock absorption ability than other MGs of the same thickness. The maximum principal strain on the maxillary incisor of MG-hs was significantly lower than that of MG-ks and MG-sw, while no significant differences were found between MG-sw and MG-ks. Extra buffer space and hard sheet improved the shock absorption ability of MG-hs, and adequate space inside the inner layer of the MG allowed orthodontic tooth movement over time. Therefore, modifications of MG-hs are highly advantageous as they exhibit better shock absorption ability than other designs of laminated MGs. This result is consistent with the findings of Takeda et al. and Bochnig et al.^28^^,^^29^ Takeda et al. specifically stated that MGs with hard inserts show significantly greater buffer capacity for shock absorption than the conventional MGs.^28^ Bochnig et al. stated that PET-G in the space between the teeth and MG significantly prevents tooth deflection by absorbing maximum forces from the impact object.^29^ Incorporating a buffer space in the MG not only leads to better shock absorption ability but also decreases the total thickness and weight of the material. Insertion of a hard material and ensuring space between the MG and teeth and/or appliance was more effective than other methods of fabricating custom-made MGs.

The control load was established considering the resistance of human bones and teeth to impact and the level of competition at which the MG would be used. Among the human facial bones, the impact tolerance of the maxilla is extremely weak. We referred the previous reports of average fracture load and the minimum tolerance of human facial bones, especially the zygoma and maxilla, which ranges from 660 N to 1700 N,^30^^,^^31^ which is almost equal to the control load applied in this study (approximately 1600 N). For this reason, we used the method with a free-falling object (500-g) on a vertical rod^22^ from a height of 5 cm for the application of the impact load.

In this study, polyolefin MG sheets were used instead of the more-commonly used ethylene vinyl acetate co-polymer (EVA) because polyolefin sheets exhibit higher adhesive strength than EVA.^32^ Custom-made MGs for patients/athletes with malocclusion undergoing orthodontic treatment should cover up to maxillary second molars on both sides, which was the protocol followed in this study. This extension not only leads to better retentive properties but also ensures efficient absorption and/or distribution of traumatic forces.^33^ Moreover, in orthodontically treated cases, inclusion of the distal-most bonded or banded molars in the MGs has been previously recommended.^18^

Shock absorption abilities of MGs have been analyzed using different types of impact objects and sensors.^22^^,^^33^,^34^ The shock absorption properties of MGs vary depending on the type of the impact object (e.g., steel ball, baseball, softball, field hockey ball, ice hockey puck, cricket ball, wooden baseball bat, etc.) and type of the sensor (e.g., strain gauge, load cell, accelerometer, etc.). However, measurements of the shock absorption abilities of MGs through an *in vitro* study can directly quantify the shock absorption ability, as the force (from a pendulum, drop ball, or piston) is measured on a transducer from a strain gauge attached beneath the MG.^35^^,^^36^ We used the strain-gauge system to measure the ability of shock absorption because of its ease of operation and data interpretation.

A limitation of this *in vitro* study was that it was performed using brackets simulated by AP blocks. Additional studies comparing actual brackets of different dimensions, with 0.18 inch and 0.22 inch slots, from different manufacturers are required, as sizes of the brackets vary according to manufacturers. Further trials using different materials from different manufacturers in patients or athletes undergoing orthodontic treatment for different types of malocclusions are warranted. Future research should also be conducted to investigate the type and amounts of adjustments required in MGs before and after tooth movement over the period of orthodontic treatment.

MG-hs showed higher ability of shock absorption, and provided increased protection against dental injuries. Insertion of a hard material and ensuring space between the MG and teeth and/or appliance was more effective than other methods of fabricating custom-made MGs to prevent sports-related traumatic dental injuries in athletes undergoing orthodontic treatment.

## Declaration of competing interest

The authors declare no conflicts of interest associated with this manuscript.
